# Supplementary material for: Orally administered Chinese herbal therapy to assist post-surgical recovery for chronic rhinosinusitis—A systematic review and meta-analysis
Source: PLoS One. 2023 Oct 5;18(10):e0292138. doi: 10.1371/journal.pone.0292138 (PMC10553817; doi:10.1371/journal.pone.0292138)
Supplement: S1 File — Including: Funnel plot of results for LK at EoT; Databases that were searched and PubMed search terms for CHM for RS; Ingredients of the CHM interventions, syndrome, manufacturer, and funding sources; Main ingredients of the Chinese herbal interventions: All studies; Main ingredients of the Chinese herbal interventions: CRSwNP studies; Main ingredients of the Chinese herbal interventions: CRSsNP studies; Risk of bias judgements for included studies; SNOT 20: Meta-analysis results for chronic rhinosinusitis post-surgery; VAS-TNS: Meta-analysis results for chronic rhinosinusitis post-surgery; VAS-IS: Meta-analysis results for chronic rhinosinusitis post-surgery; LM: Meta-analysis results for chronic rhinosinusitis post-surgery; LK: Meta-analysis results for chronic rhinosinusitis post-surgery; MTT: Meta-analysis results for chronic rhinosinusitis post-surgery; MTR: Meta-analysis results for chronic rhinosinusitis post-surgery; MC: Meta-analysis results for chronic rhinosinusitis post-surgery; Details of reported adverse events from included studies; GRADE assessments for each outcome measure. (DOC) [file pone.0292138.s002.doc]

**S1 Table. Additional data**

**Orally administered Chinese herbal therapy to assist post-surgical recovery for chronic rhinosinusitis: A systematic review and meta-analysis**

**List of additional data**

S1.1 Fig. Funnel plot of results for LK at EoT

S1.1 Table. Databases that were searched and PubMed search terms for CHM for RS

S1.2 Table. Ingredients of the CHM intervention, syndrome, manufacturer and funding sources

S1.3.1 Table. Main ingredients of the Chinese herbal interventions: All studies

S1.3.2 Table. Main ingredients of the Chinese herbal interventions: CRSwNP studies

S1.3.3 Table. Main ingredients of the Chinese herbal interventions: CRSsNP studies

S1.4 Table. Risk of bias judgements for included studies

S1.5 Table. SNOT 20: Meta-analysis results for chronic rhinosinusitis post-surgery

S1.6 Table. VAS-TNS: Meta-analysis results for chronic rhinosinusitis post-surgery

S1.7 Table. VAS-IS: Meta-analysis results for chronic rhinosinusitis post-surgery

S1.8 Table. LM: Meta-analysis results for chronic rhinosinusitis post-surgery

S1.9 Table. LK: Meta-analysis results for chronic rhinosinusitis post-surgery

S1.10 Table. MTT: Meta-analysis results for chronic rhinosinusitis post-surgery

S1.11 Table. MTR: Meta-analysis results for chronic rhinosinusitis post-surgery

S1.12 Table. MC: Meta-analysis results for chronic rhinosinusitis post-surgery

S1.13 Table. Details of reported adverse events from included studies

S1.14 Table. GRADE assessments for each outcome measure


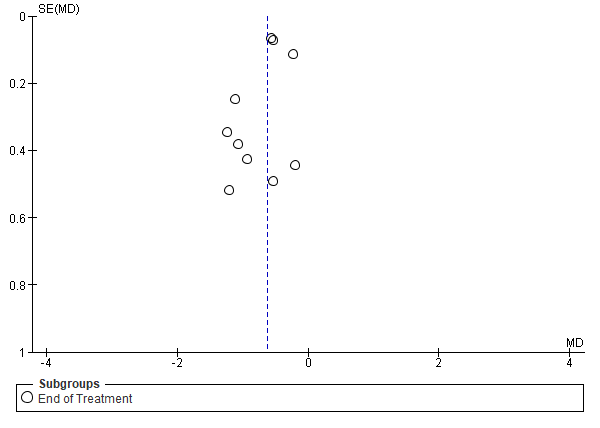


**S1.1 Fig. Funnel plot of results for LK at EoT.** Abbreviations: LK: Lund-Kennedy endoscopic score; MD, mean difference.

**S1.1 Table. Databases that were searched and PubMed search terms for CHM for RS.**

| **Databases and other resources that were searched** |
| --- |
| Searches were conducted for clinical trials assessing the effects of CHMs in the management of RS. Databases included: PubMed, Cochrane Central Register of Controlled Trials, EMBASE, AMED, CINAHL, Chinese Biomedicine, China Network Knowledge Infrastructure (CNKI), Wanfang Database and Chinese Scientific Journals Database).  In addition, targeted searches of the following sources were conducted: Australia New Zealand Clinical Trial Registry (ANZCTR); Chinese Clinical Trial Registry (ChiCTR) 中国临床试验注册中心; EU Clinical Trials Register (EU-CTR); ClinicalTrials.gov; Web of Science; ProQuest Central; and reference lists in retrieved papers. |
| **PubMed search blocks** |
| Search Terms [All Fields]  Group 1: Disorder  ‘sinusitis’[MeSH Terms] OR ‘sinusitis’[All Fields] OR rhinosinusitis [All Fields] OR rhino-sinusitis [All Fields] OR ‘nose’[MeSH Terms] OR ‘nose’[All Fields] OR ‘rhino’[All Fields] AND (‘sinusitis’[MeSH Terms] OR ‘sinusitis’[All Fields]  Group 2: Intervention  Traditional Chinese Medicine OR Chinese Traditional Medicine OR Chinese Herbal Drugs OR Chinese Drugs, Plant OR Medicine, Traditional OR Ethnopharmacology OR Ethnomedicine OR Ethnobotany OR Medicine, Kampo OR Kanpo OR TCM OR Medicine, Ayurvedic OR Phytotherapy OR Herbology OR Plants, Medicinal OR Plant Preparation OR Plant Extract OR Plants, Medicine OR Materia Medica OR Single Prescription OR Herbs OR Chinese Medicine Herb OR Herbal Medicine  Group 3: Study type  ‘randomized controlled trial’[pt] OR ‘controlled clinical trial’[pt] OR ‘randomized’[tiab] OR ‘placebo’[tiab] OR ‘drug therapy’[sh] OR ‘randomly’[tiab] OR ‘trial’[tiab] OR ‘groups’[tiab]  Combined: Group 1 AND Group 2 AND Group 3 |

Abbreviations: RS: rhinosinusitis; CHM: Chinese herbal medicine.

**S1.2 Table. Ingredients of the CHM intervention, syndrome, manufacturer and funding sources.**

| **Study ID (diagnostic criteria); (funding)** | **CHM intervention; syndrome; manufacturer** | **Ingredients of CHMs: Latin binomial (Chinese name in pinyin)**^2^ |
| --- | --- | --- |
| Chen WM 2013 (1); (NA) | *Sheng ling bai zhu san* (参苓白术散) SLBZS, decoction; NS; NS. | *Codonopsis pilosula* (Franch.) Nannf (dang shen), *Atractylodes macrocephala* Koidz (bai zhu), *Poria cocos* (Schw.) Wolf (fu ling), *Dolichos lablab* L (bai bian dou), *Dioscorea panthaica* Prain et Burk (shan yao), *Nelumbo nucifera* Gaertn (lian zi), *Angelica dahurica* (Fisch. ex Hoffm.) Benth. et Hook. f (bai zhi), *Coix lacryma-jobi* L. *var. mayuen* (Roman.) Stapf (yi yi ren), *Magnolia biondii* Pamp (xin yi hua) |
| Fan ZJ 2013 (1); (1) | CHM with no name; decoction; NS; NS. | *Houttuynia cordata* Thunb (yu xing cao), *Alisma orientalis* (Sam.) Juzep (ze xie), *Angelica sinensis* (Oliv.) Diels (dang gui), *Angelica dahurica* (Fisch. ex Hoffm.) Benth. et Hook. f (bai zhi), *Gentiana manshurica* Kitage (long dan cao), *Magnolia biondii* Pamp (xin yi hua), *Pogostemon cablin* (Blanco) Benth (huo xiang), *Ligusticum chuanxiong* Hort (chuan xiong), *Gardenia jasminoides* Elli (zhi zi), *Chrysanthemum morifolium* Ramat (ju hua), *Scutellaria baicalensis* Georgi (huang qin), *Glycyrrhiza uralensis* Fisch (gan cao) |
| Fu SW 2015 (17); (NA) | *Huo dan wan* (霍胆丸) HDW, pills; NS; Guangzhou Wanglaoji Pharmaceutical Company Limited, China. State medical permit no: Z44023699 (广州王老吉药业股份有限公司, 国药准字Z44023699). | *Pogostemon cablin* (Blanco) Benth (huo xiang), *Sus scrofadomestica* Brisson (zhu dan fen), Hydrated magnesium silicate (hua shi) |
| Gao Y 2022 (2); (2) | *Bi yan ning ke li* (鼻炎宁颗粒) BYNKL; NS; Jilin Aodong Yanbian Pharmaceutical Company Limited, China. State medical permit no: Z22022357 (吉林敖东延边药业股份有限公司,国药准字Z22022357). | *Polistes olivaceous* (DeGeer) Paper wasp nest (bi feng chao pi) |
| Li YX 2014 (3); (NA) | CHM with no name, decoction; Cold and damp coagulation & damp-heat steaming (寒湿凝聚, 湿热熏蒸); NS. | *Xanthium sibiricum* Patr (cang er zi), *Angelica dahurica* (Fisch. ex Hoffm.) Benth. et Hook. f (bai zhi), *Ligusticum chuanxiong* Hort (chuan xiong), *Asarum heterotropoides* Fr. Schmidt *var. mandshuricum* (Maxim) Kitag (xi xin), *Bupleurum chinense* DC (chai hu), *Magnolia biondii* Pamp (xin yi), *Astragalus membranaceus* (Fisch.) Bge. *var.* *mongholicus* (Bge.) Hsiao (huang qi), *Gleditsia sinensis* Lam (zao jiao ci), *Glycyrrhiza uralensis* Fisch (gan cao) |
| Liu H 2016 (4); (NA) | *Jian pi hua zhuo tong qiao fang* (健脾化浊通窍方) JPHZTQF, decoction; NS; NS. | *Astragalus membranaceus* (Fisch.) Bge. *var. mongholicus* (Bge.) Hsiao (huang qi), *Poria cocos* (Schw.) Wolf (fu ling), *Coix lacryma-jobi* L. *var. mayuen* (Roman.) Stapf (yi yi ren), *Atractylodes macrocephala* Koidz (bai zhu), *Paeonia lactiflora* Pall (bai shao), *Trichosanthes kirilowii* Maxim (tian hua fen), *Amomum villosum* Lour (sha ren), *Citrus reticulata* Blanco (chen pi), *Platycodon grandiflorum* (Jacq.) A. DC. (jie geng), *Eupatorium fortunei* Turcz (pei lan), *Pogostemon cablin* (Blanco) Benth (huo xiang), *Cimicifuga heracleifolia* Kom (sheng ma), *Angelica dahurica* (Fisch. ex Hoffm.) Benth. et Hook. f. (bai zhi), *Acorus tatarinowii* Schott (shi chang pu), *Glycyrrhiza uralensis* Fisch (gan cao) |
| Mou S 2015 (5,6); (NA) | *Shu du tang* (枢度汤) SDT, decoction; Stagnant heat in the Gallbladder (胆腑郁热); prepared on site at Affiliated Hospital of Chengdu Traditional Chinese Medicine University, China (本方药由成都中医药大学附属医院自煎. 煎服取汁 200 mL, 每袋100 mL, 每日２袋, 早晚温服). | *Bupleurum chinense* DC (chai hu), *Paeonia lactiflora* Pall (bai shao), *Trichosanthes kirilowii* Maxim (gua lou), *Astragalus membranaceus* (Fisch.) Bge. *var.* *mongholicus* (Bge.) Hsiao (huang qi), *Angelica dahurica* (Fisch. ex Hoffm.) Benth. et Hook. f (bai zhi), *Pheretima aspergillum* (E. Perrier) (di long), *Platycodon grandiflorum* (Jacq.) A. DC (jie geng), *Scutellaria baicalensis* Georgi (huang qin), *Poria cocos* (Schw.) Wolf (fu ling), *Acorus tatarinowii* Schott (shi chang pu), *Citrus aurantium* L (zhi ke), *Ligusticum chuanxiong* Hort (chuan xiong), *Angelica sinensis* (Oliv.) Diels (dang gui), *Paeonia lactiflora* Pall (chi shao, *Salvia miltiorrhiza* Bge (dan shen), *Atractylodes macrocephala* Koidz (bai zhu) |
| Shu YY 2013 (7,8); (NA) | *Wu wei xiao du yin* (五味消毒饮) WWXDY, decoction; Heat accumulation in the Lung meridian (肺经蕴热型; prepared on site at Shuguang Hospital Affiliated to Shanghai University of Traditional Chinese Medicine, China. | *Lonicera japonica* Thunb (jin yin hua), Chrysanthemum indicum L (ye ju hua), *Taraxacum mongolicum* Hand. -Mazz (pu gong ying), *Nervilia fordii* (Hance) Schltr (zi bei tian kui), *Viola yedoensis* Makino (zi hua di ding), *Magnolia biondii* Pamp (xin yi hua), *Astragalus membranaceus* (Fisch.) Bge. *var.* *mongholicus* (Bge.) Hsiao (huang qi), *Ligusticum chuanxiong* Hort (chuan xiong), *Angelica dahurica* (Fisch. ex Hoffm.) Benth. et Hook. f (bai zhi) |
| Tan GD 2011 (1,9); (NA) | CHM with no name, decoction; Lung Spleen *qi* deficiency (肺脾气虚); NS. | *Astragalus membranaceus* (Fisch.) Bge. *var.* *mongholicus* (Bge.) Hsiao (huang qi), *Codonopsis pilosula* (Franch.) Nannf (dang shen), *Atractylodes macrocephala* Koidz (bai zhu), *Saposhnikovia divaricata (*Turcz.) Schischk (fang feng), *Xanthium sibiricum* Patr (cang er zi), *Magnolia biondii* Pamp (xin yi hua), *Angelica dahurica* (Fisch. ex Hoffm.) Benth. et Hook. f (bai zhi), *Mentha haplocalyx* Briq (bo he), *Lonicera japonica* Thunb (jin yin hua), *Trichosanthes kirilowii* Maxim (tian hua fen), *Benincasa hispida* (Thunb.) Cogn (dong gua ren), *Glycyrrhiza uralensis* Fisch (gan cao) |
| Wang Y 2017 (10,11); (NA) | *Bi yuan tong qiao ke li* (鼻渊通窍颗粒) BYTQKL, pills; NS; NS. | *Scutellaria baicalensis* Georgi (huang qin), *Ephedra sinica* Stapf (ma huang), *Mentha haplocalyx* Briq (bo he), *Forsythia suspensa* (Thunb.) Vahl (lian qiao) |
| Wang YJ 2015 (17); (NA) | *Bi yuan tong qiao ke li* (鼻渊通窍颗粒) BYTQKL, pills; NS; NS. | *Magnolia biondii* Pamp (xin yi), *Angelica dahurica* (Fisch. ex Hoffm.) Benth. et Hook. f (bai zhi), *Xanthium sibiricum* Patr (cang er zi), *Ephedra sinica* Stapf (ma huang), *Ligusticum sinense* Oliv (gao ben), *Forsythia suspensa* (Thunb.) Vahl (lian qiao), *Scutellaria baicalensis* Georgi (huang qin), *Trichosanthes kirilowii* Maxim (tian hua fen), *Mentha haplocalyx* Briq (bo he), *Salvia miltiorrhiza* Bge (dan shen), *Rehmannia glutinosa* Libosch (di huang), *Chrysanthemum indicum* L (ye ju hua), *Poria cocos* (Schw.) Wolf (fu ling), *Glycyrrhiza uralensis* Fisch (gan cao)^1^ |
| Zeng YS 2016 (2,9); (3) | *Huang qin hua shi tang* (黄芩滑石汤) HQHST, decoction; Spleen-Stomach dampness heat (脾胃湿热); prepared at the Second People’s Hospital affiliated to Fujian University of Traditional Chinese Medicine, China (所服用的中药汤剂由医院中药房统一代煎). | *Scutellaria baicalensis* Georgi (huang qin), Hydrated magnesium silicate (hua shi), *Poria cocos* (Schw.) Wolf (fu ling), *Acorus tatarinowii* Schott (shi chang pu), *Amomum krarvanh* Pierre ex Gagnep (bai dou kou), *Polyporus umbellatus* (Pers.) Fires (zhu ling), *Xanthium sibiricum* Patr (cang er zi), *Angelica dahurica* (Fisch. ex Hoffm.) Benth. et Hook. f (bai zhi), *Magnolia biondii* Pamp (xin yi), *Glycyrrhiza uralensis* Fisch (gan cao) |
| Zeng YS 2021 (12,13); (4) | *Huang qin hua shi tang* (黄芩滑石汤) HQHST, decoction; Spleen–Stomach dampness heat (脾胃湿热); prepared at the Second People’s Hospital affiliated to Fujian University of Traditional Chinese Medicine, China (所服用的中药汤剂由医院中药房统一代煎). | *Scutellaria baicalensis* Georgi (huang qin), Hydrated magnesium silicate (hua shi), *Poria cocos* (Schw.) Wolf (fu ling), *Acorus tatarinowii* Schott (shi chang pu), *Amomum krarvanh* Pierre ex Gagnep (bai dou kou), *Polyporus umbellatus* (Pers.) Fires (zhu ling), *Xanthium sibiricum* Patr (cang er zi), *Angelica dahurica* (Fisch. ex Hoffm.) Benth. et Hook. f (bai zhi), *Magnolia biondii* Pamp (xin yi), *Glycyrrhiza uralensis* Fisch (gan cao) |
| Zhang EQ 2018 (2); (NA) | CHM with no name, decoction; NS; NS. | *Astragalus membranaceus* (Fisch.) Bge. *var.* *mongholicus* (Bge.) Hsiao (huang qi), *Xanthium sibiricum* Patr (cang er zi), *Saposhnikovia divaricata (*Turcz.) Schischk (fang feng), *Carthamus tinctorius* L (hong hua), *Salvia miltiorrhiza* Bge (dan shen), *Glycyrrhiza uralensis* Fisch (gan cao) |
| Zhang J 2016 (10,11); (NA) | *Bi yuan tong qiao ke li* (鼻渊通窍颗粒) BYTQKL, pills; Stagnant heat in the Gallbladder (胆腑郁热); Shandong New Times Pharmaceutical Co, Ltd. (山东新时代药业有限公司). | *Scutellaria baicalensis* Georgi (huang qin), *Mentha haplocalyx* Briq (bo he), *Ephedra sinica* Stapf (ma huang), *Magnolia biondii* Pamp (xin yi), *Xanthium sibiricum* Patr (cang er zi), *Salvia miltiorrhiza* Bge (dan shen), *Rehmannia glutinosa* Libosch (sheng di huang), *Trichosanthes kirilowii* Maxim (tian hua fen), *Poria cocos* (Schw.) Wolf (fu ling) |
| Zhang JY 2019 (12); (NA) | *Xin qian gan ju tang* (辛前甘桔汤) XQGJT, decoction; NS; prepared on site at Shuguang Hospital Affiliated to Shanghai University of Traditional Chinese Medicine, China (中药制剂由医院制剂室提供). | *Magnolia biondii* Pamp (xin yi), *Saposhnikovia divaricata (*Turcz.) Schischk (fang feng), *Peucedanum praeruptorum* Dunn (qian hu), *Trichosanthes kirilowii* Maxim (tian hua fen), *Coix lacryma-jobi* L. *var. mayuen* (Roman.) Stapf (yi yi ren), *Platycodon grandiflorum* (Jacq.) A. DC (jie geng), *Glycyrrhiza uralensis* Fisch (gan cao) |
| Zhang R (2,9); 2021 (5) | *Qing re li shi qu yu tang* (清热利湿祛瘀汤) QRLSQYT, decoction; Spleen-Stomach dampness heat (脾胃湿热); prepared on site at Nanjing Integrated Traditional Chinese and Western Medicine Hospital, China (中药制剂由医院中药房提供). | *Xanthium sibiricum* Patr (cang er zi), *Angelica dahurica* (Fisch. ex Hoffm.) Benth. et Hook. f (bai zhi), *Magnolia biondii* Pamp (xin yi), *Ligusticum chuanxiong* Hort (chuan xiong), *Angelica sinensis* (Oliv.) Diels (dang gui), *Houttuynia cordata* Thunb (yu xing cao), *Alisma orientalis* (Sam.) Juzep (ze xie), *Scutellaria baicalensis* Georgi (huang qin), *Chrysanthemum morifolium* Ramat (ju hua), *Pogostemon cablin* (Blanco) Benth (huo xiang), *Gentiana manshurica* Kitage (long dan cao), *Gardenia jasminoides* Elli (zhi zi), *Glycyrrhiza uralensis* Fisch (gan cao) |
| Zhang WQ (2,14); 2020 (NA) | *Shen ling bai zhu san* (参苓白术散) SLBZS, decoction; Spleen *qi* deficiency (脾气虚弱); prepared at the Second People’s Hospital affiliated to Fujian University of Traditional Chinese Medicine, China (所服用的中药汤剂由医院中药房统一代煎). | *Codonopsis pilosula* (Franch.) Nannf (dang shen), *Atractylodes macrocephala* Koidz (bai zhu), *Poria cocos* (Schw.) Wolf (fu ling), *Glycyrrhiza uralensis* Fisch (gan cao), *Dolichos lablab* L (bai bian dou), *Dioscorea panthaica* Prain et Burk (shan yao), *Coix lacryma-jobi* L. *var. mayuen* (Roman.) Stapf (yi yi ren), *Platycodon grandiflorum* (Jacq.) A. DC (jie geng), *Amomum villosum* Lour (sha ren), *Citrus reticulata* Blanco (chen pi), *Magnolia biondii* Pamp (xin yi), *Ligusticum chuanxiong* Hort (chuan xiong) |
| Zheng XR 2017 (15); (NA) | *Bi yan kang pian* (鼻炎康片) BYKP, tablets; NS; Foshan Dezhong Pharmaceutical Industry Limited Company, China. State medical permit no: Z44021580 (佛山德众药业有限公司, 国药准字 Z44021580). | *Pogostemon cablin* (Blanco) Benth (huo xiang), *Sus scrofadomestica* Brisson (zhu dan fen), *Angelica sinensis* (Oliv.) Diels (dang gui), *Scutellaria baicalensis* Georgi (huang qin), Chrysanthemum indicum L (ye ju hua), *Ephedra sinica* Stapf (ma huang), *Mentha haplocalyx* Briq (bo he), *Xanthium sibiricum* Patr (cang er zi) |
| Zhou ML 2016 (2,16); (NA) | *Wen fei zhi liu dan* (温肺止流丹) WFZLD, decoction; Lung *qi* deficiency with cold (肺气虚寒); prepared at the Second People’s Hospital affiliated to Fujian University of Traditional Chinese Medicine, China (所服用的中药汤剂由医院中药房统一代煎). | *Astragalus membranaceus* (Fisch.) Bge. *var.* *mongholicus* (Bge.) Hsiao (huang qi), *Codonopsis pilosula* (Franch.) Nannf (dang shen), *Atractylodes macrocephala* Koidz (bai zhu), *Terminalia chebula* Retz (he zi), *Platycodon grandiflorum* (Jacq.) A. DC (jie geng), *Schizonepeta tenuifolia* Briq (jing jie), *Xanthium sibiricum* Patr (cang er zi), *Magnolia biondii* Pamp (xin yi hua), *Asarum heterotropoides* Fr. Schmidt *var. mandshuricum* (Maxim) Kitag (xi xin), *Angelica dahurica* (Fisch. ex Hoffm.) Benth. et Hook. f. (bai zhi), *Houttuynia cordata* Thunb (yu xing cao), *Glycyrrhiza uralensis* Fisch (gan cao) |
| Zhu HH 2014 (7); (6) | *Qing bi tang* (清鼻汤) QBT, decoction; NS; NS. | *Scutellaria baicalensis* Georgi (huang qin), *Angelica dahurica* (Fisch. ex Hoffm.) Benth. et Hook. f. (bai zhi), *Glycyrrhiza uralensis* Fisch (gan cao), *Gentiana manshurica* Kitage (long dan cao), *Ephedra sinica* Stapf (ma huang), *Lonicera japonica* Thunb (jin yin hua). *Imperata cylindrica* Beauv. *var. major* (Nees) C. E. Hubb (bai mao gen), *Cinnamomum* *cassia* Presl (gui zhi), *Bupleurum chinense* DC (chai hu), *Xanthium sibiricum* Patr (cang er zi), *Magnolia biondii* Pamp (xin yi hua), *Diospyros kaki* Thunb (shi chang pu), *Curcuma wenyujin* Y.H. Chen et C. Ling (yu jin), *Asarum heterotropoides* Fr. Schmidt *var. mandshuricum* (Maxim) Kitag (xi xin), *Houttuynia cordata* Thunb (yu xing cao) |

Notes: 1. no ingredients were listed in the article. These ingredients are from URL: [https://baike.baidu.com/item/%E9%BC%BB%E6%B8%8A%E9%80%9A%E7%AA%8D%E9%A2%97%E7%B2%92/6466215](https://baike.baidu.com/item/鼻渊通窍颗粒/6466215);

2. Scientific names are based on: Chinese Pharmacopoeia Commission. Pharmacopoeia of the People’s Republic of China [Zhong Hua Ren Min Gong He Guo Yao Dian 中华人民共和国药典]. Beijing: China Medical Science Press [中国医药科技出版社]; 2020.

Abbreviations: CHM: Chinese herbal medicine; NA: not applicable; NS: not specified.

**Diagnostic criteria:**

1. Otorhinolaryngology Branch of Chinese Medical Association, editorial board of Chinese Journal of Otolaryngology. Clinical classification and staging of chronic sinusitis nasal breath and evaluation criteria for the efficacy of endoscopic sinus surgery (1997, Haikou) [S]. Chinese Journal of Otorhinolaryngology, 1998, 33(3): 134. 中华医学会耳鼻咽喉科学分会, 中华耳鼻咽喉科杂志编委会. 慢性鼻窦炎鼻息内临床分型分期及内窥镜鼻窦手术疗效评定标准(1997, 海口)[S]. 中华耳鼻咽喉科杂志, 1998, 33(3):134.

2. Rhinology Group of Editorial Committee of Chinese Journal of Otolaryngology-Head and Neck Surgery, Rhinology Group of Otorhinolaryngology-Head and Neck Surgery Branch of Chinese Medical Association. Guidelines for the diagnosis and treatment of chronic rhinosinusitis (2012, Kunming [J]. Chinese Journal of Otolaryngology Head and Neck Surgery, 2013 48( 2): 92-94. 中华耳鼻咽喉头颈外科杂志编辑委员会鼻科组, 中华医学会耳鼻咽喉头颈外科学分会鼻科学组. 慢性鼻-鼻窦炎诊断和治疗指南( 2012年, 昆明［J］中华耳鼻咽喉头颈外科杂志, 2013 48( 2): 92-94.

3. Otorhinolaryngology with Integrated Traditional Chinese and Western Medicine. 中西医结合耳鼻喉科学

4. Guiding Principles for Clinical Research of New Drugs of Traditional Chinese Medicine, Issued by the Ministry of Health of the People's Republic of China. 1997, Volume 3, 168-169. 中华人民共和国卫生部制定发布《中药新药临床研究指导原则》第3 辑, 1997, 168-169

5. Fokkens W, Lund V, Mullol J; European Position Paper on Rhinosinusitis and Nasal Polyps group. European position paper on rhinosinusitis and nasal polyps 2007. Rhinol Suppl. 2007;20:1-136. PMID: 17844873.

6. Guideline for Diagnosis and Treatment of Common Diseases of E.N.T in TCM. Chinese Association of Traditional Chinese Medicine. 中医耳鼻咽喉科常见病诊疗指南. 中华中医药学会.

7. Editorial Board of Journal of Head and Neck Surgery Chinese Otolaryngology. Guidelines for diagnosis and treatment of chronic rhinosinusitis (2008 Nanchang)[J]. Chinese Journal of Otorhinolaryngology-Head and Neck Surgery, 2009, 44(1): 6. 头颈外科杂志编委会中华耳鼻咽喉. 慢性鼻-鼻窦炎诊断和治疗指南(2008南昌)[J]. 中华耳鼻咽喉头颈外科杂志, 2009, 44(1):6.

8. Wang Yongqin. Otorhinolaryngology of Traditional Chinese Medicine, People’s Health Publishing House, 2011, second edition. 王永钦, 中医耳鼻咽喉口腔学, 人民卫生出版社,, 2011年, 第二版.

9. Wang Shizhen. Traditional Chinese Medicine Otolaryngology. Beijing: China Press of Traditional Chinese Medicine, 2003. 王士贞. 中医耳鼻咽喉科学, 北京: 中国中医药出版社, 2003.

10. Bu Guoyi. Rhinology [M]. 2 editions. Shanghai: Shanghai Science and Technology Press, 2000.卜国铱. 鼻科学[M]. 2版．上海: 上海科学技术出版社, 2000.

11. Wang Dejian. Traditional Chinese Medicine Otorhinolaryngology Stomatology [M]. Beijing: People’s Medical Publishing House, 1994. 王德鉴. 中医耳鼻咽喉口腔科学[M]. 北京: 人民卫生出版社, 1994.

12. Guidelines for Diagnosis and Treatment of Chronic Nasal Sinusitis in China. Rhinology Group, Otorhinolaryngology-Head and Neck Surgery Branch, Chinese Medical Association. (2018). 中华医学会耳鼻咽喉头颈外科学分会鼻科学组, 中国慢性鼻窦炎诊断和治疗指南

13. Liu Peng. Otorhinolaryngology of Traditional Chinese Medicine, 2016. 刘蓬. 中医耳鼻咽喉科学.

14. Ruan Yan. Otorhinolaryngology of Traditional Chinese Medicine. People’s Medical Publishing House, 2012. 阮岩. 中医耳鼻咽喉科学, 人民卫 生出版社, 2012.

15. Editorial Board of Chinese Journal of Otorhinolaryngology-Head and Neck Surgery. Guidelines for Diagnosis and Treatment of Chronic Rhinosinusitis[J]. Chinese Journal of Clinicians, 2010. 中华耳鼻咽喉头颈外科杂志编委会. 慢性鼻-鼻窦炎诊断和治疗指南[J]. 中国临床医生杂志, 2010.

16. Wang Shizhen. Clinical Research of Otorhinolaryngology of Traditional Chinese Medicine. 2009. 王士贞. 中医耳鼻咽喉科临床研究. 2009.

17. Unclear

**Funding sources**:

1. 基金项目: 广东省中山市医学科学技术研究基金项目, 项目编号: 201005;
2. 基金项目: 山西省医疗器械智能准确检测项目(2020SYS14); 山西省卫生厅项目(2021146);
3. 基金项目: 福建省中青年教师教育科研项目(A类) (JA14166);
4. 基金项目: 福建省中青年教师教育科研项目( A 类) (JAT170262);
5. 基金项目: 2018 年度南京卫生科技发展专项资金项目 (YKK18170);
6. 河南省卫生厅医学科技攻关计划项目 (201003026).

**S1.3.1 Table. Main ingredients of the Chinese herbal interventions: All studies**

| **Scientific name (name in Chinese pin yin)** | **No. CHM** **interventions^1^** |
| --- | --- |
| *Magnolia biondii* Pamp (xin yi 辛夷) | 15 |
| *Angelica dahurica* (Fisch. ex Hoffm.) Benth. et Hook. f (bai zhi 白芷) | 14 |
| *Glycyrrhiza uralensis* Fisch (gan cao 甘草) | 13 |
| *Xanthium sibiricum* Patr (cang er zi 苍耳子) | 12 |
| *Scutellaria baicalensis* Georgi (huang qin 黄芩) | 11 |
| *Poria cocos* (Schw.) Wolf (fu ling 茯苓) | 7 |
| *Astragalus membranaceus* (Fisch.) Bge. var. *mongholicus* (Bge.) Hsiao (huang qi 黄芪) | 7 |
| *Ligusticum chuanxiong* Hort (chuan xiong 川芎) | 6 |
| *Mentha haplocalyx* Briq (bo he 薄荷) | 6 |
| *Atractylodes macrocephala* Koidz (bai zhu 白术) | 6 |

Note: 1. Based on formulae in 22 test groups.

Abbreviations: CHM: Chinese herbal medicine; No.: number.

**S1.3.2 Table. Main ingredients of the Chinese herbal interventions: CRSwNP studies**

| **Scientific name (name in Chinese pin yin)** | **No. CHM** **interventions^1^** |
| --- | --- |
| *Magnolia biondii* Pamp (xin yi 辛夷) | 6 |
| *Angelica dahurica* (Fisch. ex Hoffm.) Benth. et Hook. f (bai zhi 白芷) | 6 |
| *Glycyrrhiza uralensis* Fisch (gan cao 甘草) | 4 |
| *Xanthium sibiricum* Patr (cang er zi 苍耳子) | 4 |
| *Scutellaria baicalensis* Georgi (huang qin 黄芩) | 3 |
| *Poria cocos* (Schw.) Wolf (fu ling 茯苓) | 4 |
| *Ligusticum chuanxiong* Hort (chuan xiong 川芎) | 4 |
| *Atractylodes macrocephala* Koidz (bai zhu 白术) | 4 |
| *Scutellaria baicalensis* Georgi (huang qin 黄芩) | 3 |
| *Astragalus membranaceus* (Fisch.) Bge. var. *mongholicus* (Bge.) Hsiao (huang qi 黄芪) | 3 |
| *Codonopsis pilosula* (Franch.) Nannf. (dang shen 党参) | 3 |

Note: 1. Based on formulae in 7 test groups.

Abbreviations: CHM: Chinese herbal medicine; No.: number.

**S1.3.3 Table. Main ingredients of the Chinese herbal interventions: CRSsNP studies**

| **Scientific name (name in Chinese pin yin)** | **No. CHM** **interventions^1^** |
| --- | --- |
| *Scutellaria baicalensis* Georgi (huang qin 黄芩) | 4 |
| *Xanthium sibiricum* Patr (cang er zi 苍耳子) | 3 |
| *Mentha haplocalyx* Briq (bo he 薄荷) | 3 |
| *Pogostemon cablin* (Blanco) Benth. or *Agastache rugosa* (Fisch. & Mey.) O. Ktze. (huo xiang 藿香) | 3 |
| *Ephedra sinica* Stapf (ma huang 麻黄) | 3 |

Note: 1. Based on formulae in 6 test groups.

Abbreviations: CHM: Chinese herbal medicine; No.: number.

**S1.4 Table. Risk of bias assessments for included studies**

| **Ref No.** | **Included studies** | **Risk of Bias Categories** | | | | | | |
| --- | --- | --- | --- | --- | --- | --- | --- | --- |
|  | **Study ID** **(First author year)** | **SG** | **AC** | **BPt** | **BPn** | **BOA** | **IOD** | **SOR** |
| 62 | Chen WM 2013 | U | U | H | H | H | L | U |
| 63 | Fan ZJ 2013 | U | U | H | H | H | L | U |
| 47 | Fu SW 2015 | U | U | H | H | H | L | U |
| 50 | Gao Y 2022 | L | U | U | U | U | L | U |
| 52 | Li YX 2014 | U | U | H | H | H | L | U |
| 64 | Liu H 2016 | U | U | H | H | H | L | U |
| 51 | Mou S 2015 | L | U | H | H | H | L | U |
| 55 | Shu YY 2013 | L | U | H | H | H | L | U |
| 53 | Tan GD 2011 | U | U | H | H | H | L | U |
| 56 | Wang Y 2017 | L | U | H | H | H | L | U |
| 65 | Wang YJ 2015 | U | U | H | H | H | L | U |
| 66 | Zeng YS 2016 | U | U | H | H | H | L | U |
| 67 | Zeng YS 2021 | U | U | H | H | H | L | U |
| 48 | Zhang EQ 2018 | U | U | H | H | H | L | U |
| 57 | Zhang J 2016 | L | U | H | H | H | L | U |
| 58 | Zhang JY 2019 | L | U | H | H | H | L | U |
| 59 | Zhang R 2021 | L | U | H | H | H | L | U |
| 60 | Zhang WQ 2020 | L | U | H | H | H | L | U |
| 68 | Zheng XR 2017 | U | U | H | H | H | L | U |
| 61 | Zhou ML 2016 | L | U | H | H | H | L | U |
| 49 | Zhu HH 2014 | L | U | H | H | H | L | U |
|  | **Number ‘Low’ (%)** | 10 L (47.6) | 0 L (0) | 0 L (0) | 0 L (0) | 0 L (0) | 21 L (100) | 0 L (0) |

Abbreviations: AC: allocation concealment; BOA: blinding of outcome assessment; BPn: blinding of personnel; BPt: blinding of participants; H: high risk; IOD: incomplete outcome data; L: low risk; SG: sequence generation; SOR: selective outcome reporting; U: unclear risk or no information specified.

**S1.5 Table. SNOT-20: Meta-analysis results for chronic rhinosinusitis post-surgery**

| **Study ID (First author year)** | **No. studies (No. participants: T, C) Treatment duration** | **T vs C at BL; MD [95% CI], I^2^** | **T vs C at EoT/FU; MD [95% CI], I^2^** |
| --- | --- | --- | --- |
| Li YX 2014 | 1 (30,30) 4wks | 5 items: −0.06 [−1.10, 0.98];  Total score: 0.15 [−2.31, 2.61]. | EoT 5 items: −0.98 [−1.93, −0.03]*  EoT total score: −1.11 [−3.04, 0.82]  FU 6 mths ps:  total score: −3.37 [−5.10, −1.64]*^1^  5 items: −0.75 [−1.63, 0.13]  FU 12 mths ps  total score: −0.84 [−2.19, 0.51]  5 items: −0.16 [−0.97, 0.65]  FU 24 mths ps  total score: −0.67 [−1.74, 0.40]  5 items: −0.86 [−1.69, −0.03]* |
| Zhang EQ 2018 | 1 (51,51) 15d | 0.14 [−2.20, 2.48] | −6.50 [−8.01, −4.99]* |
| **Pool EoT** total score, all  [Li YX 2014, Zhang EQ 2018] | 2 (81,81) 15d–4wks | 0.14 [−1.55, 1.84], 0% | −3.84 [−9.12, 1.44], 95% |
| Fu SW 2015 (FU) | 1 (45,45) 12mths | BL: NA | EoT: NA;  FU 6 mths ps −3.40 [−4.85, −1.95]* |
| **Pool FU** all, total score  [Fu SW 2015, Li YX 2014] | 2 (67,66) longest FU 6–24 mths ps | BL: NA | −1.99 [−4.66, 0.68], 89% |
| Sens 1. FU total score same timepoint  [Fu SW 2015, Li YX 2014] | 2 (73,74) 6 mths ps | BL: NA | −3.39 [−4.50, −2.28]*, 0% |

* significant difference.

Abbreviations: BL: baseline; C: control group; CI: confidence interval; d: days; EoT: end of treatment; FU: follow up; I^2^: index of heterogeneity; MD: mean difference; mths: months; No.: number; NA: not applicable; ps: post-surgery; sens: sensitivity analysis; SNOT: Sino-Nasal Outcome Test; T: treatment group; wks: weeks.

Note: 1. This was due to the control group getting worse, not the CHM group getting better.

**S1.6 Table. VAS-TNS: Meta-analysis results for chronic rhinosinusitis post-surgery**

| **Study ID (First author year)** | **No. studies (No. participants: T, C) Treatment duration** | **T vs C at BL; MD [95% CI], I^2^** | **T vs C at EoT/FU; MD [95% CI], I^2^** |
| --- | --- | --- | --- |
| Zheng XR 2017 | 1 (39,39) 2mths | 0.02 [−1.22, 1.26] | EoT: −1.81 [−2.45, −1.17]* |
| Zhou ML 2016 | 1 (30,30) 4wks | 0.40 [−0.23, 1.03] | EoT: −0.90 [−1.71, −0.09]* |
| Zeng YS 2021 | 1 (30,30) 6wks | NA | EoT: −0.23 [−0.47, 0.01]  FU 8wks ps: −0.56 [−0.70, −0.42]* |
| Zhang JY 2019 | 1 (83,83) 12wks | 0.60 [−0.85, 2.05] | EoT: 0.20 [−0.91, 1.31] |
| Zhang WQ 2020 | 1 (30,30) 4wks | 0.43 [−0.16, 1.02] | EoT: −1.36 [−2.43, −0.29]* |
| Mou S 2015 | 1 (20,20) 4wks | 0.30 [−0.84, 1.44] | EoT: −1.20 [−2.19, −0.21]* (8 wks ps) |
| Chen WM 2013 | 1 (25,25) 3mths | NA | EoT: −1.60 [−2.06, −1.14]* |
| Zeng YS 2016 | 1 (36,36) 6wks | NA | EoT: −0.31 [−0.52, −0.10]*  FU 8wks ps: −0.58 [−0.71, −0.45]* |
| **Pool: EoT** all  [Zheng XR 2017, Zhou ML 2016, Zeng YS 2021, Zhang JY 2019, Zhang WQ 2020, Mou S 2015, Chen WM 2013, Zeng YS 2016] | 8 (293,293) 4–12 wks | NA | −0.89 [−1.36, −0.41]*, 87% |
| Sens 1. all with no sig BL (excl NA)  [Zheng XR 2017, Zhou ML 2016, Zhang JY 2019, Zhang WQ 2020, Mou S 2015] | 5 (202,202) 4–12 wks | 0.38 [0.01, 0.75]*, 0% | −1.08 [−1.72, −0.44]*, 61% |
| Sens 2. all 4 wks treatment, no sig BL  [Zhou ML 2016, Zhang WQ 2020, Mou S 2015] | 3 (80,80) 4wks | 0.30 [−0.84, 1.44], 0% | −1.11 [−1.65, −0.57]*, 0% |
| Sens 3. *Shen ling bai zhu san* [Chen WM 2013, Zhang WQ 2020] | 2 (55,55) 4wks – 3mths | NA | −1.56 [−1.99, −1.14]*, 0% |
| Sens 4. *Huang qin hua shi tang* [Zeng YS 2016, Zeng YS 2021] | 2 (66,66) 6wks | NA | −0.27 [−0.43, −0.11]*, 0% |
| Sens 5. CRSwNP [Chen WM 2013, Mou S 2015, Zeng YS 2021, Zhang WQ 2020] | 4 (105, 105) 4wks – 3mths | NA | −1.06 [−1.95, −0.17]*, 90% |
| Sens 6. CRSsNP [Zeng YS 2016, Zheng XR 2017] | 2 (75,75) 6wks – 2mths | NA | −1.03 [−2.50, 0.44], 95% |
| Sens 7. Spleen-Stomach dampness heat 脾胃湿热 [Zeng YS 2016, Zeng YS 2021]^1^ | 2 (66,66) 6wks | NA | −0.27 [−0.43, −0.11]*, 0% |
| **Pool: longest FU**  [Zeng YS 2016, Zeng YS 2021]^1^ | 2 (66,66) 6wks | NA | −0.57 [−0.67, −0.47]*, 0% (FU 8wks ps) |

* significant difference.

Abbreviations: BL: baseline; C: control group; CI: confidence interval; CRSsNP: CRS without nasal polyps; CRSwNP: CRS with nasal polyps; EoT: end of treatment; excl: excluding; I^2^: index of heterogeneity; MD: mean difference; mths: months; No.: number; NA: not applicable; ps: post-surgery; sens: sensitivity analysis; sig: significant; T: treatment group; VAS-TNS: visual analogue scale scores for total nasal symptoms; wks: weeks.

Note: 1. Both studies used the same formula *Huang qin hua shi tang* 黄芩滑石汤.

**S1.7 Table. VAS-IS: Meta-analysis results for chronic rhinosinusitis post-surgery**

| **Study ID (First author year)** | **No. studies (No. participants: T, C) Treatment duration** | **T vs C at BL; MD [95% CI], I^2^** | **T vs C at EoT/FU; MD [95% CI], I^2^** |
| --- | --- | --- | --- |
| Shu YY 2013 | 1 (30,30) 2wks | Congestion −0.34 [−0.84, 0.16] | Congestion −0.90 [−1.68, −0.12]* |
|  |  | Discharge 0.47 [−0.60, 1.54] | Discharge −0.90 [−1.62, −0.18]* |
|  |  | Dizziness/headache −0.33 [−1.28, 0.62] | Dizziness/headache −0.76 [−1.34, −0.18]* |
|  |  | Olfactory decline 0.07 [−1.22, 1.36] | Olfactory decline −1.34 [−2.58, −0.10]* |
| Li YX 2014 | 1 (30,30) 4wks | Congestion −0.03 [−0.74, 0.68] | Congestion −0.79 [−1.43, −0.15]*  FU 6mths ps −0.70 [−1.30, −0.10]*  FU 12mths ps −0.69 [−1.08, −0.30]*  FU 24mths ps −0.52 [−0.90, −0.14]* |
|  |  | Discharge −1.76 [−2.57, −0.95]* | Discharge −0.85 [−1.55, −0.15]*  FU 6mths ps −0.43 [−1.03, 0.17]*  FU 12mths ps −0.72 [−1.16, −0.28]*  FU 24mths ps −0.18 [−0.65, 0.29]* |
|  |  | Headache/ facial pain −0.28 [−1.39, 0.83] | Headache/ facial pain −1.05 [−1.68, −0.42]*  FU 6mths ps −0.03 [−0.65, 0.59]*  FU 12mths ps −0.31 [−0.77, 0.15]*  FU 24mths ps −0.34 [−0.82, 0.14]* |
|  |  | Olfactory decline 0.12 [−0.83, 1.07] | Olfactory decline −1.43 [−2.25, −0.61]*  FU 6mths ps −0.10 [−0.51, 0.31]*  FU 12mths ps 0.09 [−0.36, 0.54]*  FU 24mths ps −0.20 [−0.60, 0.20]* |
| Wang Y 2017 | 1 (40,40) 4wks | Pain 0.02 [−0.67, 0.71] | Pain −0.81 [−1.03, −0.59]* |
| Zhang J 2016 | 1 (50,50) 4wks | Pain −0.03 [−0.28, 0.22] | Pain −0.94 [−1.10, −0.78]* |
| **Pool: Congestion EoT** [Shu YY 2013, Li YX 2014] | 2 (60,60) 2–4wks EoT | Congestion −0.24 [−0.65, 0.17], 0% | Congestion −0.83 [−1.33, −0.34]*, 0% |
| **Pool: Discharge EoT** [Shu YY 2013, Li YX 2014] | 2 (60,60) 2–4wks EoT | Discharge −0.67 [−2.86, 1.51]*, 91% | Discharge −0.87 [−1.38, −0.37]*, 0% |
| **Pool: Olfactory decline EoT** [Shu YY 2013, Li YX 2014] | 2 (60,60) 2–4wks EoT | Olfactory decline 0.10 [−0.66, 0.87] | Olfactory decline −1.40 [−2.09, −0.72]*, 0% |
| **Pool: Pain EoT** [Li YX 2014, Wang Y 2017, Zhang J 2016] | 3 (120,120) 4wks EoT | Pain −0.04 [−0.26, 0.19], 0% | Pain −0.90 [−1.03, −0.78]*, 0% |
| Sens 1. Stagnant heat in the Gallbladder 胆腑郁热VAS-IS (pain) [Wang Y 2017, Zhang J 2016 ]^1^ | 2 (90,90) 4wks | Pain −0.02 [−0.26, 0.21], 0% | Pain −0.89 [−1.02, −0.77]*, 0% |

* significant difference.

Abbreviations: BL: baseline; C: control group; CI: confidence interval; EoT: end of treatment; FU: follow up; I^2^: index of heterogeneity; MD: mean difference; mths: months; No.: number; ps: post-surgery; T: treatment group; VAS-IS: visual analogue scale scores for individual symptoms; wks: weeks.

Note: 1. Both studies used the same formula *Bi yuan tong qiao ke li* 鼻渊通窍颗粒, both CRSsNP.

**S1.8 Table. LM: Meta-analysis results for chronic rhinosinusitis post-surgery**

| **Study ID (First author year)** | **No. studies (No. participants: T, C) Treatment duration** | **T vs C at BL; MD [95% CI], I^2^** | **T vs C at EoT/FU; MD [95% CI], I^2^** |
| --- | --- | --- | --- |
| Zeng YS 2016 | 1 (36,36) 6wks | 0.23 [−0.74, 1.20] | EoT: NA  FU 8 wks ps: −0.95 [−1.61, −0.29]* |
| Zhang JY 2019 | 1 (83,83) 12wks | 0.44 [−0.53, 1.41] | EoT: NA  FU 12 mth ps: −0.53 [−0.87, −0.19] |
| **Pool: FU** all  [Zeng YS 2016, Zhang JY 2019] | 2 (119,109) 8wks – 12mths ps | 0.34 [−0.35, 1.02], 0% | −0.64 [−1.00, −0.28]*, 18% |

* significant difference.

Abbreviations: BL: baseline; C: control group; CI: confidence interval; EoT: end of treatment; FU: follow up; I^2^: index of heterogeneity; LM: Lund-Mackay computed tomography (CT) score; MD: mean difference; mths: months; No.: number; NA: not applicable; ps: post-surgery; T: treatment group; wks: weeks.

**S1.9 Table. LK: Meta-analysis results for chronic rhinosinusitis post-surgery**

| **Study ID (First author year)** | **No. studies (No. participants: T, C) Treatment duration** | **T vs C at BL; MD [95% CI], I^2^** | **T vs C at EoT/FU; MD [95% CI], I^2^** |
| --- | --- | --- | --- |
| Shu YY 2013 | 1 (30,30) 2wks | 0.03 [−0.85, 0.91] | EoT: −1.24 [−1.92, −0.56]*  FU 12 wks ps: −1.47 [−2.25, −0.69]* |
| Zhang EQ 2018 | 1 (51,51) 15d | BL: NA | EoT: NA  FU 12mths ps: −0.60 [−0.93, −0.27]* |
| Zhou ML 2016 | 1 (30,30) 4wks | 0.13 [−0.43, 0.69] | EoT: −0.93 [−1.77, −0.09]* |
| Zhu HH 2014 | 1 (96,98) 8wks | 0.17 [−0.59, 0.93] | EoT: −1.12 [−1.61, −0.63]*  FU 12 wks ps: −2.48 [−2.82, −2.14] |
| Fu SW 2015 | 1 (45,45) 2mths | BL: NA | EoT: −0.20 [−1.07, 0.67] |
| Zeng YS 2016 | 1 (36,36) 6wks | BL: NA | EoT: −0.56 [−0.69, −0.43]*  FU 8 wks ps: −0.21 [−0.33, −0.09]* |
| Li YX 2014 | 1 (30,30) 4wks | −0.11 [−0.88, 0.66] | EoT: −0.54 [−1.50, 0.42]  FU 6mths ps: −1.92 [−2.74, −1.10]*  FU 12mths ps: −1.11 [−1.36, −0.86]*  FU 24mths ps: −0.90 [−1.16, −0.64]* |
| Zeng YS 2021 | 1 (30,30) 6wks | BL: NA | EoT: −0.54 [−0.68, −0.40]*  FU 8 wks ps: −0.24 [−0.37, −0.11]* |
| Zhang JY 2019 | 1 (83,83) 12wks | −0.11 [−0.47, 0.25] | EoT: −0.23 [−0.45, −0.01]* |
| Zhang WQ 2020 | 1 (30,30) 4wks | 0.07 [−0.19, 0.33] | EoT: −1.07 [−1.82, −0.32]* |
| Mou S 2015 | 1 (20,20) 4wks | 0.80 [−0.17, 1.77] | EoT: −1.20 [−2.21, −0.19]* |
| **Pool: EoT** all [Shu YY 2013, Zhou ML 2016, Zhu HH 2014, Fu SW 2015, Zeng YS 2016, Li YX 2014, Zeng YS 2021, Zhang JY 2019, Zhang WQ 2020, Mou S 2015] | 10 (430,432) 2–12 wks | BL: NA | −0.63 [−0.82, −0.44]*, 59% |
| Sens 1. all with no sig BL (excl NA)  [Shu YY 2013, Zhou ML 2016, Zhu HH 2014, Li YX 2014, Zhang JY 2019, Zhang WQ 2020, Mou S 2015] | 7 (319,321) 2–12 wks | 0.05 [−0.13, 0.23], 0% | −0.86 [−1.30, −0.43]*, 72% |
| Sens 2. all 4 wks treatment, no sig BL [Zhou ML 2016, Li YX 2014, Zhang WQ 2020, Mou S 2015] | 4 (110,110) 4wks | 0.10 [−0.12, 0.32], 0% | −0.95 [−1.38, −0.51]*, 0% |
| Sens 3. *Huang qin hua shi tang* [Zeng YS 2016, Zeng YS 2021] | 2 (66,66) 6wks | NA | −0.55 [−0.65, −0.45]*, 0% |
| Sens 4. CRSwNP [Li YX 2014, Mou S 2015, Zeng YS 2021, Zhang WQ 2020] | 4 (110,110) 4–6 wks | 0.12 [−0.20, 0.44], 15% | −0.62 [−0.86, −0.39]*, 11% |
| Sens 5. CRSsNP [Fu SW 2015, Zeng YS 2016] | 2 (81,81) 6 wks – 2 mths | NA | −0.55 [−0.68, −0.42]*, 0% |
| Sens 6. Spleen-Stomach dampness heat 脾胃湿热 [Zeng YS 2016, Zeng YS 2021]^1^ | 2 (66,66) 6wks | NA | −0.55 [−0.65, −0.45]*, 0% |
| **Pool: FU** all, longest [Shu YY 2013, Zhang EQ 2018, Zhu HH 2014, Zeng YS 2016, Li YX 2014, Zeng YS 2021] | 6 (265,266) 8 wks – 24 mths ps | NA | −0.95 [−1.47, −0.42]*, 97% |
| Sens 1. FU all with no sig BL (excl NA) [Shu YY 2013, Zhu HH 2014, Li YX 2014] | 3 (156/158) 12 wks – 24 mths ps | 0.03 [−0.43, 0.49], 0% | −1.62 [−2.78, −0.46]*, 96% |
| Sens 2. FU 12 wks ps  [Shu YY 2013, Zhu HH 2014] | 2 (126,128) 12 wks ps | 0.11 [−0.47, 0.69], 0% | −2.04 [−3.02, −1.06]*, 81% |

* significant difference.

Abbreviations: BL: baseline; C: control group; CI: confidence interval; CRSsNP: CRS without nasal polyps; CRSwNP: CRS with nasal polyps; EoT: end of treatment; excl: excluding; FU: follow up; I^2^: index of heterogeneity; LK: Lund-Kennedy Endoscopic score; MD: mean difference; mths: months; No.: number; NA: not applicable; ps: post-surgery; sens: sensitivity analysis; sig: significant; T: treatment group; wks: weeks.

Note: 1. Both studies used the same formula *Huang qin hua shi tang* 黄芩滑石汤.

**S1.10 Table. MTT: Meta-analysis results for chronic rhinosinusitis post-surgery**

| **Study ID (First author year)** | **No. studies (No. participants: T, C) Treatment duration** | **T vs C at BL; MD [95% CI] minutes, I^2^** | **T vs C at EoT/FU; MD [95% CI] minutes, I^2^** |
| --- | --- | --- | --- |
| Wang Y 2017 | 1 (40,40) 4wks | 0.04 [−1.15, 1.23] | EoT: −5.42 [−6.28, −4.56]* |
| Zhang J 2016 | 1 (50,50) 4wks | −0.02 [−0.53, 0.49] | EoT: −5.09 [−5.50, −4.68]* |
| Liu H 2016 | 1 (32,32) 8wks | 0.03 [−0.65, 0.71] | EoT: −2.85 [−3.44, −2.26]* |
| Zhang R 2021 | 1 (46,46) 12wks | −0.52 [−1.25, 0.21] | EoT: −3.73 [−4.26, −3.20]* |
| Wang YJ 2015 | 1 (75,75) 2wks | 0.06 [−0.38, 0.50] | EoT: −3.10 [−3.46, −2.74]*  FU 4wks ps: −6.02 [−6.41, −5.63]*  FU 6wks ps: −5.01 [−5.36, −4.66]*  FU 8wks ps: −5.02 [−5.35, −4.69]* |
| **Pool: EoT** all  [Wang Y 2017, Zhang J 2016, Liu H 2016, Zhang R 2021, Wang YJ 2015] | 5 (243,243) 2–12wks | −0.05 [−0.32, 0.22], 0% | −4.02 [−5.00, −3.03]*, 95% |
| Sens 1. all 4 wks treatment  [Wang Y 2017, Zhang J 2016] | 2 (90,90) 4wks | −0.01 [−0.48, 0.46], 0% | −5.15 [−5.52, −4.78]*, 0% |
| Sens 2. *Bi yuan tong qiao ke li* [Wang YJ 2015, Zhang J 2016, Wang Y 2017] | 3 (165,165) 2–4wks | 0.03 [−0.29, 0.35], 0% | −4.51 [−6.06, −2.96]*, 97% |
| Sens 3. CRSsNP [Liu H 2016, Wang Y 2017, Zhang J 2016] | 3 (122,122) 2–4wks | 0.00 [−0.39, 0.39], 0% | −4.44 [−6.02, −2.87]*, 95% |
| Sens 4. Stagnant heat in the Gallbladder 胆腑郁热 [Zhang J 2016, Wang Y 2017]^1^ | 2 (90,90) 4wks | −0.01 [−0.48, 0.46], 0% | −5.15 [−5.52, −4.78]*, 0% |

* significant difference.

Abbreviations: BL: baseline; C: control group; CRSsNP: CRS without nasal polyps; CI: confidence interval; EoT: end of treatment; FU: follow up; I^2^: index of heterogeneity; MD: mean difference; mths: months; MTT: mucociliary transport time; No.: number; ps: post-surgery; sens: sensitivity analysis; T: treatment group; wks: weeks.

Note: 1. Both studies used the same formula *Bi yuan tong qiao ke li* 鼻渊通窍颗粒, both CRSsNP.

**S1.11 Table. MTR: Meta-analysis results for chronic rhinosinusitis post-surgery**

| **Study ID (First author year)** | **No. studies (No. participants: T, C) Treatment duration** | **T vs C at BL; MD [95% CI] mm/min, I^2^** | **T vs C at EoT/FU; MD [95% CI] mm/min, I^2^** |
| --- | --- | --- | --- |
| Chen WM 2013 | 1 (25,25) 3mths | NA | EoT: 2.08 [1.37, 2.79]* |
| Tan GD 2011 | 1 (50,50) 3mths | 0.10 [−0.14, 0.34] | EoT: 1.27 [0.91, 1.63]*  FU 6 mths ps 1.19 [0.80, 1.58]* |
| Wang Y 2017 | 1 (40,40) 4wks | 0.05 [−0.16, 0.26] | EoT: 2.20 [1.91, 2.49]* |
| Zhang J 2016 | 1 (50,50) 4wks | −0.04 [−0.19, 0.11] | EoT: 2.84 [2.63, 3.05]* |
| Zhu HH 2014 | 1 (96,98) 8wks | −0.06 [−0.23, 0.11] | EoT: NA  FU 12 wks ps 0.99 [0.70, 1.28]* |
| Liu H 2016 | 1 (32,32) 8wks | 0.01 [−0.20, 0.22] | EoT: 1.50 [1.20, 1.80]* |
| Zeng YS 2021 | 1 (30,30) 6wks | −0.07 [−0.20, 0.06] | EoT: 0.31 [0.17, 0.45]*  FU 8 wks ps 0.46 [0.33, 0.59]* |
| Zhang R 2021 | 1 (46,46) 12wks | 0.11 [−0.13, 0.35] | EoT: 0.70 [0.45, 0.95]* |
| Fan ZJ 2013 | 1 (41,41) 3mths | 0.00 [−0.13, 0.13] | EoT: 1.40 [1.07, 1.73]*  FU 6 mths ps 0.60 [0.19, 1.01]* |
| Wang YJ 2015 | 1 (75,75) 2wks | 0.04 [−0.09, 0.17] | EoT: 1.83 [1.66, 2.00]*  FU 4wks ps 2.57 [2.42, 2.72]*  FU 6wks ps 2.77 [2.62, 2.92]*  FU 8wks ps 2.75 [2.56, 2.94]* |
| **Pool: EoT** excl Zhu HH 2014  [Chen WM 2013, Tan GD 2011, Wang Y 2017, Zhang J 2016, Liu H 2016, Zeng YS 2021, Zhang R 2021, Fan ZJ 2013, Wang YJ 2015] | 9 (389,389) 2–12 wks | NA | 1.56 [0.93, 2.20]*, 98% |
| Sens 1. all with no sig BL (excl Chen 2013)  [Tan GD 2011, Wang Y 2017, Zhang J 2016, Liu H 2016, Zeng YS 2021, Zhang R 2021, Fan ZJ 2013, Wang YJ 2015] | 8 (364,364) 2–12 wks | 0.00 [−0.05, 0.06], 0% | 1.51 [0.83, 2.18]*, 99% |
| Sens 2. 12 wks EoT  [Chen WM 2013, Tan GD 2011, Zhang R 2021, Fan ZJ 2013] | 4 (162,162) 12wks | NA | 1.29 [0.80, 1.78]*, 86% |
| Sens 3. 12 wks EoT, all with no sig BL (excl Chen 2013)  [Tan GD 2011, Zhang R 2021, Fan ZJ 2013] | 3 (137,137) 12wks | 0.04 [−0.06, 0.14], 0% | 1.11 [0.65, 1.57]*, 85% |
| Sens 4. 4 wks, EoT all with no sig BL  [Wang Y 2017, Zhang J 2016] | 2 (90,90) 4wks | −0.01 [−0.13, 0.11], 0% | 2.53 [1.90, 3.16]*, 92% |
| Sens 5. *Bi yuan tong qiao ke li* [Wang YJ 2015, Zhang J 2016, Wang Y 2017] | 3 (165,165) 2–4wks | 0.01 [−0.08, 0.10], 0% | 2.29 [1.64, 2.94]*, 96% |
| Sens 6. CRSwNP [Chen WM 2013, Tan GD 2011, Zeng YS 2021] | 3 (105,105) 6 wks – 3 mths | NA | 1.17 [0.22, 2.11]*, 95% |
| Sens 7. CRSsNP [Liu H 2016, Wang Y 2017, Zhang J 2016] | 3 (122,122) 4–8wks | −0.01 [−0.11, 0.10], 0% | 2.19 [1.40, 2.97]*, 96% |
| Sens 8. Stagnant heat in the Gallbladder 胆腑郁热 [Wang Y 2017, Zhang J 2016]^1^ | 2 (90,90) 4wks | −0.01 [−0.13, 0.11], 0% | 2.53 [1.90, 3.16]*, 92% |
| Sens 9. Spleen-Stomach dampness heat 脾胃湿热 [Zeng YS 2021, Zhang R 2021] | 2 (76,76) 4wks | −0.01 [−0.18, 0.16], 38% | 0.49 [0.11, 0.87]* ,86% |
| **Pool: FU** all, longest FU, all no sig BL  [Tan GD 2011, Zhu HH 2014, Zeng YS 2021, Fan ZJ 2013, Wang YJ 2015] | 5 (292,253) 8 wks – 6 mths ps | −0.01 [−0.08, 0.06], 0% | 1.20 [0.14, 2.26]*, 99% |
| Sens 1. FU 6 mths ps  [Tan GD 2011, Fan ZJ 2013] | 2 (91,91) 6 mths ps | 0.02 [−0.09, 0.14], 0% | 0.90 [0.32, 1.48]*, 76% |

* significant difference.

Abbreviations: BL: baseline; C: control group; CI: confidence interval; CRSsNP: CRS without nasal polyps; CRSwNP: CRS with nasal polyps; EoT: end of treatment; excl: excluding; FU: follow up; I^2^: index of heterogeneity; MD: mean difference; mths: months; mm/min: millimetres per minute; MTR: mucociliary transport rate: No.: number; NA: not applicable; ps: post-surgery; sens: sensitivity analysis; sig: significant; T: treatment group; wks: weeks.

Note: 1. Both studies used the same formula *Bi yuan tong qiao ke li* 鼻渊通窍颗粒, both CRSsNP.

**S1.12 Table. MC percentage: Meta-analysis results for chronic rhinosinusitis post-surgery**

| **Study ID (First author year)** | **No. studies (No. participants: T, C) Treatment duration** | **T vs C at BL; MD [95% CI], I^2^** | **T vs C at EoT; MD [95% CI], I^2^** |
| --- | --- | --- | --- |
| Wang Y 2017 | 1 (40,40) 4wks | 0.15 [−2.10, 2.40] | EoT: 9.84 [6.75, 12.93] |
| Zhang J 2016 | 1 (50,50) 4wks | −0.05 [−2.51, 2.41] | EoT: 9.37 [6.58, 12.16]* |
| Liu H 2016 | 1 (32,32) 8wks | 0.02 [−3.35, 3.39] | EoT: 9.63 [6.16, 13.10]* |
| Zhang R 2021 | 1 (46,46) 12wks | −0.44 [−3.64, 2.76] | EoT: 8.09 [5.30, 10.88]* |
| Wang YJ 2015 | 1 (75,75) 2wks | 0.04 [−1.99, 2.07] | EoT: 2.20 [0.19, 4.21]*  FU 4wks ps: 8.09 [5.93, 10.25]*  FU 6wks ps: 7.18 [4.97, 9.39]*  FU 8wks ps: 8.86 [6.59, 11.13]* |
| **Pool**: **EoT** all, no sig BL  [Wang Y 2017, Zhang J 2016, Liu H 2016, Zhang R 2021, Wang YJ 2015] | 5 (243,243) 2–12 wks | −0.01 [−1.14, 1.11], 0% | 7.71 [4.31, 11.12]*, 87% |
| Sens 1. 4 wks duration  [Wang Y 2017, Zhang J 2016] | 2 (90,90) 4wks | 0.06 [−1.60, 1.72], 0% | 9.58 [7.51, 11.65]*, 0% |
| Sens 2. *Bi yuan tong qiao ke li* [Wang YJ 2015, Zhang J 2016, Wang Y 2017] | 3 (165,165) 2–4wks | 0.05 [−1.23, 1.34], 0% | 7.04 [1.71, 12.38]*, 92% |
| Sens 3. CRSsNP [Liu H 2016, Zhang J 2016, Wang Y 2017] | 3 (122,122) 2–4wks | 0.05 [−1.44, 1.54], 0% | 9.59 [7.82, 11.37]*, 0% |
| Sens 4. Stagnant heat in the Gallbladder 胆腑郁热 [Zhang J 2016, Wang Y 2017]^1^ | 2 (90,90) 4wks | 0.06 [−1.60, 1.72], 0% | 9.58 [7.51, 11.65]*, 0% |

* significant difference.

Abbreviations: BL: baseline; C: control group; CI: confidence interval; CRSsNP: CRS without nasal polyps; EoT: end of treatment; FU: follow up; I^2^: index of heterogeneity; MC: mucociliary clearance; MD: mean difference; No.: number; ps: post-surgery; sens: sensitivity analysis; sig: significant; T: treatment group; wks: weeks.

Note: 1. Both studies used the same formula *Bi yuan tong qiao ke li* 鼻渊通窍颗粒, both CRSsNP.

**S1.13 Table. Details of reported adverse events from included studies**

| **Study ID (First author year); duration; participants (T, C)** | **Test group (T)** | **Control group (C) PT** | **Adverse events** |
| --- | --- | --- | --- |
| Mou S 2015; 4wks; (20,20) | Oral SDT 4 weeks after surgery | Routine antibiotic treatment after surgery plus oral placebo 4 weeks after surgery | T: 0;  C: 2 (dizzy = 1, nausea = 1). |
| Zheng XR 2017; 2mths; (39,39) | Oral BYKP plus PT | Routine antibiotic treatment after surgery | T: 5 (nausea and vomiting = 3, diarrhoea = 1, tired = 1);  C: 4 (nausea and vomiting = 2, diarrhoea = 1, tired = 1). |
| Gao Y 2022; 3mths; (79,79) | Oral BYNKL | Budesonide nasal spray | T: 5 (dry nose = 3, dizzy and headache = 1, indigestion = 1); C:12 (dry nose = 7, dizzy and headache = 3, indigestion = 2). |
| Gao Y 2022; 3mths; (79,79) | Oral BYNKL plus PT | Budesonide nasal spray | T:2 (dry nose = 2;  C:12 (dry nose = 7, dizzy and headache = 3, indigestion = 2). |
| Fu SW 2015; Shu YY 2013; Wang YJ 2015; Zhang J 2016; Zhang R 2021; Zhang WQ 2020; Zhou ML 2016 | 7 studies of various CHMs | Various PTs | T: 0;  C: 0 |
| Chen WM 2013; Fan ZJ 2013; Li YX 2014; Liu H 2016; Tan GD 2011; Wang Y 2017; Zeng YS 2016; Zeng YS 2021; Zhang EQ 2018; Zhang JY 2019; Zhu HH 2014 | 11 studies of various CHMs | Various PTs | Adverse events were not reported. |

Abbreviations: C: control group; CHM: Chinese herbal medicine; mths: months; PT: pharmacotherapy; T: test group; wks: weeks.

**S1.14 Table. GRADE assessments for each outcome measure**

| **Outcome (timepoint)** | **No. RCTs** | **No. participants** | **MD [95% CI], I^2^** | **GRADE** |
| --- | --- | --- | --- | --- |
| SNOT-20 (EoT) | 2 | 162 | −3.84 [−9.12, 1.44], 95% | Very low ^1–4^ |
| SNOT-20 (FU, 6mths ps) | 2 | 147 | −3.39 [−4.50, −2.28]*, 0% | Very low ^1,2,4^ |
| VAS-TNS (EoT) | 8 | 576 | −0.89 [−1.36, −0.41]*, 87% | Very low ^1,3,4^ |
| LM (longest FU) | 2 | 228 | −0.64 [−1.00, −0.28]*, 18% | Very low ^1,2,4^ |
| LK (EoT) | 10 | 862 | −0.63 [−0.82, −0.44]*, 59% *p* = 0.009 sig | Very low ^1,3,4^ |
| LK (longest FU) | 6 | 531 | −0.95 [−1.47, −0.42]*, 97% | Very low ^1,3,4^ |
| LK (FU, 12wks ps) | 2 | 254 | −2.04 [−3.02, −1.06]*, 81% | Very low ^1–4^ |
| MTT (EoT) | 5 | 486 | −4.02 [−5.00, −3.03]*, 95% | Very low ^1,3,4^ |
| MTR (EoT) | 9 | 778 | 1.56 [0.93, 2.20]*, 98% | Very low ^1,3,4^ |
| MTR (longest FU) | 5 | 545 | 1.20 [0.14, 2.26]*, 99% | Very low ^1,3,4^ |
| MTR (FU, 6mths ps) | 2 | 182 | 0.90 [0.32, 1.48]*, 76% | Very low ^1–4^ |
| MC (EoT) | 5 | 486 | 7.71 [4.31, 11.12]*, 87% | Very low ^1,3,4^ |
| MC (EoT, 4wks) | 2 | 180 | 9.58 [7.51, 11.65]*, 0% | Very low ^1,2,4^ |

Abbreviations: CI: confidence interval; EoT, end of treatment; FU, follow up; I^2^: index of heterogeneity; LK: Lund-Kennedy Endoscopic score; LM: Lund-Mackay computed tomography (CT) score; MC: mucociliary clearance %; MD, mean difference; mths: months; MTR: mucociliary transport rate; MTT: mucociliary transport time; No.: number; ps, post-surgery; SD, standard deviation; sig: significant; RCT: randomised controlled trial; SNOT: Sino-Nasal Outcome Test; VAS-TNS: visual analogue scale scores for total nasal symptoms; wks: weeks.

Notes: 1. Rated down for lack of blinding; 2. Rated down for small sample size; 3. Rated down for heterogeneity; 4. Rated down for lack of protocol and potential selective outcome reporting.
