# Supplementary material for: Orally administered Chinese herbal therapy to assist post-surgical recovery for chronic rhinosinusitis—A systematic review and meta-analysis
Source: PLoS One. 2023 Oct 5;18(10):e0292138. doi: 10.1371/journal.pone.0292138 (PMC10553817; doi:10.1371/journal.pone.0292138)
Supplement: Attachment — Submitted filename: PONE-D-23-14802 Responses to reviewers 17 08 23.docx [file pone.0292138.s003.docx]

**PONE-D-23-14802**

Orally administered Chinese herbal therapy to assist post-surgical recovery for chronic rhinosinusitis  -  A systematic review and meta-analysis

**Responses to reviewers**

We wish to thank the reviewers for their constructive comments and suggestions. We believe that the revised manuscript has benefitted greatly from the review process.

Revisions have been made using track changes.

Line numbers refer to the final MS version without track changes.

Each of the reviewers’ comments has been numbered consecutively and responded to in order as follows:

**Reviewer #1**: Some suggestions to improve the overall quality:

**Comment 1.**
-Include only double-blind, placebo-controlled studies to ensure a rigorous assessment of the Chinese herbal interventions. This will strengthen the validity and certainty of the evidence.

**Response 1.**

Thank you for this suggestion. We had hoped to find a number of double-blind, placebo-controlled studies. However, only one study used a placebo, and it was unclear if it was double blinded or not.

We have noted this issue in the Limitations section (L595):

‘In future studies, a placebo for the oral CHM should be used to enable blinding of participants and personnel. This would strengthen the validity and certainty of the evidence.’

**Comment 2.**
- Increase the sample sizes of included studies to provide more precise effect size estimates and allow for robust meta-analyses. Around 400-500 patients per arm would be reasonable.

**Response 2.**

Thank you for this suggestion. The available studies were mostly small in size and consequently the sizes of the meta-analysis pools were below optimum.

We have noted this issue in the Limitations section (L643):

‘The relatively small sizes of the studies and the meta-analysis pools (less than 500 participants) was a concern and led to the downgrading of the evidence.’

**Comment 3.**
- Require that all included studies report adverse events in detail, to allow for a proper safety analysis of the herbal medicines. Reporting of adverse events should follow established guidelines.

**Response 3.**

Thank you for this suggestion. The reporting of AEs was deficient in the studies. We have emphasised this issue in the Limitations section and included some additional references (L 619):

‘There was a lack of safety reporting in most studies. Under-reporting of adverse drug reactions and AEs is a major issue internationally [95]. Pharmacovigilance largely depends on adequate reporting, so this is an important responsibility of clinicians and researchers to fully report all AEs according to established guidelines [96-98].’

**Comment 4.**
- Consider stratifying the meta-analyses by Chinese medicine syndrome or CRS endotype, if sufficient data are available, to determine if effects vary by subgroup. This could help explain some of the heterogeneity.

**Response 4.**

Thank you for this valuable suggestion. In the results, we have added additional sensitivity analyses for CRSwNP, CRSsNP and Chinese medicine syndrome, where possible.

In the Data analysis section we added (L185):

‘Post-hoc sensitivity analyses included presence or absence of nasal polyps and Chinese medicine syndrome.’

In the Results section, we added the results of all these additional sensitivity analyses. These were also included in the supplementary tables (S6-S12).

This was also included this issue in the Limitations (L607):

‘Overall, heterogeneity marred many of the meta-analysis results. This was somewhat reduced in the sensitivity analyses. The additional sensitivity analyses for CRSwNP and CRSsNP reduced heterogeneity for LK and MC. While those for CM syndrome reduced heterogeneity for VAS-TNS, VAS-IS pain, LK, MTT and MC. However, this was at the expense of reducing the size of meta-analysis pools.’

**Comment 5.**
- Extend the follow-up periods in included studies to determine if any benefits are sustained long-term. At least 6-12 months of follow-up would be preferable.

**Response 5.**

Thank you for this suggestion. Only a few studies had adequate follow-ups. In the limitations section we have added (L645):

‘Another limitation was relatively few studies reported FU data, and only one study reported long-term FUs. It is important to determine whether any benefits associated with the addition of CHMs to usual care are sustained long term and whether there are any effects on rates of recurrence. Future studies should report FUs of 6-12 months.’

**Comment 6.**
- Improve the introduction and cite these evidences for placebo-controlled trials of herbal medicines:

**Response 6.**

Thank you for this suggestion. In the introduction we have added a section on complementary therapies (L85):

‘Various complementary therapies also have been used for RS, including herbal medicines [13, 14], homeopathy [15-17], and acupuncture therapies [18-21]. Nevertheless, when these approaches do not provide sufficient control of symptoms, such as facial pain and headache [22], surgery can be effective [1, 3, 23, 24].’

In this section we have included:
14 Barnes J, Anderson LA, Phillipson JD. Herbal medicines. A guide for health-care professionals. 2nd ed. London: Pharmaceutical Press; 2002.

Plus another reference on herbal medicine:
13.	Taw MB, Nguyen CT, Wang MB. Complementary and integrative treatments: rhinosinusitis. Otolaryngol Clin North Am. 2013;46(3):345-66. PubMed PMID: Medline:23764814.

17	Witt CM, Lüdtke R, Baur R, Willich SN. Homeopathic medical practice: long-term results of a cohort study with 3981 patients. BMC Public Health. 2005 Nov 3;5:115. doi: 10.1186/1471-2458-5-115. PMID: 16266440; PMCID: PMC1298309.

Plus other references on homeopathy:

15.	Nayak C, Singh V, Singh VP, Oberai P, Roja V, Shitanshu SS, et al. Homeopathy in chronic sinusitis: a prospective multi-centric observational study. Homeopathy. 2012;101(2):84-91. Epub 2012/04/11. doi: 10.1016/j.homp.2012.02.002. PubMed PMID: 22487367.

16.	Zabolotnyi DI, Kneis KC, Richardson A, Rettenberger R, Heger M, Kaszkin-Bettag M, et al. Efficacy of a complex homeopathic medication (Sinfrontal) in patients with acute maxillary sinusitis: a prospective, randomized, double-blind, placebo-controlled, multicenter clinical trial. Explore (NY). 2007;3(2):98-109. Epub 2007/03/17. doi: 10.1016/j.explore.2006.12.007. PubMed PMID: 17362845.

21 Vickers AJ, Linde K. Acupuncture for chronic pain. JAMA. 2014 Sep 10;312(10):955-6.
Plus other references on acupuncture:

18.	Stavem K, Rossberg E, Larsson PG. Health-related quality of life in a trial of acupuncture, sham acupuncture and conventional treatment for chronic sinusitis. BMC Res Notes. 2008;1:37. Epub 2008/08/20. doi: 10.1186/1756-0500-1-37. PubMed PMID: 18710490; PubMed Central PMCID: PMCPMC2546421.

19.	Rossberg E, Larsson PG, Birkeflet O, Soholt LE, Stavem K. Comparison of traditional Chinese acupuncture, minimal acupuncture at non-acupoints and conventional treatment for chronic sinusitis. Complement Ther Med. 2005;13(1):4-10. doi: 10.1016/j.ctim.2005.01.002. PubMed PMID: WOS:000229650400002.

20.	Lundeberg T, Laurell G, Thomas M. Effect of acupuncture on sinus pain and experimentally induced pain. Ear Nose Throat J. 1988;67(8):565-6, 71-2, 74-5. Epub 1988/08/01. PubMed PMID: 3248531.

**Comment 7.**
- CHMs could become an important integrative treatment option for CRS, especially for patients who do not achieve complete control of their symptoms with surgery and conventional medical management alone CHMs may offer a personalized, patient-centered approach that addresses the root causes of CRS from a traditional medicine perspective. Concentrate especially for symptoms as cephalea and obstruction rhinogenic. Discuss the role of and cite doi:10.1007/s00405-021-06724-6

**Response 7.**

Thank you for this suggestion. It has been included just prior to the conclusions section (L650):

‘Following further research, CHMs could become important integrative treatment options for CRS, especially for patients who do not achieve complete control of their symptoms with surgery and conventional medical management alone. A personalised, patient-centred approach that addresses the root causes of CRS from both conventional and traditional medicine perspectives could assist in the management of difficult-to-treat conditions involving cephalea and rhinogenic obstruction [22].’

**Comment 8.**

Improve the methods with the following study for adverse event reporting guidelines:

• Edwards IR, Lindquist M, Vilhelmsen K, et al. Under-reporting of adverse drug reactions. A global perspective. Drug Saf. 1998 Apr;18(4):215-23

**Response 8.**

Thank you for this suggestion. We could not locate the above paper despite extensive searches. So, we have included another paper on the same topic by Edwards, in the method section, as follows:

64	Edwards IR. Communicating the new pharmacovigilance landscape. Drug Saf. 2012;35(12):1085-9. Epub 2012/11/10. doi: 10.1007/BF03261995. PubMed PMID: 23137149.

In the section on safety reporting in the limitations section, we have included this paper plus others:

97.	Edwards IR, Aronson JK. Adverse drug reactions: definitions, diagnosis, and management. Lancet. 2000 Oct 7;356(9237):1255-9. doi: 10.1016/S0140-6736(00)02799-9. PMID: 11072960.

98.	Farah MH, Edwards R, Lindquist M, Leon C, Shaw D. International monitoring of adverse health effects associated with herbal medicines. Pharmacoepidemiol Drug Saf. 2000;9(2):105-12. Epub 2008/11/26. doi: 10.1002/(SICI)1099-1557(200003/04)9:2<105::AID-PDS486>3.0.CO;2-2. PubMed PMID: 19025809.

Plus a paper with the same title:

96.	Hazell L, Shakir AWS. Under-Reporting of Adverse Drug Reactions. Drug Safety. 2006;29(5):385-96. doi: 10.2165/00002018-200629050-00003. PMID: 16689555.

The amendments to Limitations (L620)

‘There was a lack of safety reporting in most studies. Under-reporting of adverse drug reactions and AEs is a major issue internationally [96]. Pharmacovigilance largely depends on adequate reporting, so this is an important responsibility of clinicians and researchers to fully report all AEs according to established guidelines [64, 97, 98].’

**Comment 9.**
Discuss the role of more feasible and accessible subjective score for symptoms assessment and cite doi: 10.1007/s00405-023-07855-8.

**Response 9.**

Thank you for this suggestion. We have included this in the discussion. (L490)

‘Future studies could consider using SNOT-8 as a more feasible and accessible subjective score for symptom assessment [59].’

With the following reference:

59	La Mantia I, Ragusa M, Grigaliute E, Cocuzza S, Radulesco T, Calvo-Henriquez C, Saibene AM, Riela PM, Lechien JR, Fakhry N, Michel J, Maniaci A. Sensibility, specificity, and accuracy of the Sinonasal Outcome Test 8 (SNOT-8) in patients with chronic rhinosinusitis (CRS): a cross-sectional cohort study. Eur Arch Otorhinolaryngol. 2023 Jul;280(7):3259-3264. doi: 10.1007/s00405-023-07855-8. Epub 2023 Jan 27. PMID: 36705726.

**Comment 10.**
- Cite additional relevant literature on Chinese herbal medicine for CRS, placebo-controlled clinical trials of herbal medicines, recommendations for reporting of clinical trials of herbal interventions, and adverse event reporting guidelines.

**Response 10.**

Thank you for this suggestion. We have added additional literature to the introduction (see above), including (L114):

‘The CHMs can be administered orally and/or nasally [43, 44].’

Plus an extra reference

44 Yang Y, Sun Y, Xiang F, Zhang M, Fu L, Zhang Q. Efficacy and safety of traditional Chinese medicine nasal irrigation on chronic rhinosinusitis recovery after endoscopic sinus surgery: A protocol for a systematic review and meta-analysis. PLoS One. 2022 Aug 1;17(8):e0272371. doi: 10.1371/journal.pone.0272371. PMID: 35913939; PMCID: PMC9342793.

Added an extra section in the limitations on reporting of clinical trials (L617):

‘The Consolidated Standards of Reporting Trials (CONSORT) [94, 95] should be followed to ensure rigorous, internationally recognised methods are employed and all data are reported adequately.’

Added a section on adverse event reporting (L620):

‘There was a lack of safety reporting in most studies. Under-reporting of adverse drug reactions and AEs is a major issue internationally [96].’

**Comment 11.**
- Downgrade the certainty of the evidence to "Very Low" for all outcomes due to the major limitations of the included studies, including lack of blinding and high risk of bias.

**Response 11.**

Thank you for this suggestion.

All GRADEs have been changed to very low (L464)

‘The GRADE assessments (S14 Table) were downgraded for each outcome by one GRADE due to the lack of blinding in the studies and by one GRADE for lack of a protocol and consequent risk of selective outcome reporting. For SNOT-20 (FU), LM (FU), MC (EoT) and MC (EoT, 4 weeks), the assessments were further downgraded for small sample size (less than 400) to very low certainty. For VAS-TNS (EoT), LK (EoT), LK (longest FU), MTT (EoT), MTR (EoT) and MTR (longest FU), the sample sizes were over 400 participants, but there was significant heterogeneity in the meta-analysis result. Therefore, these were each downgraded to very low certainty. For SNOT-20 (EoT), LK (FU, 12 weeks post-surgery), and MTR (FU, 6 months post-surgery), each was downgraded to very low certainty.’

See also Supplementary S14 Table

**Other responses**

Thank you for the suggestions for additional references. The following have been added:

22	Maniaci A, Merlino F, Cocuzza S, Iannella G, Vicini C, Cammaroto G, Lechien JR, Calvo-Henriquez C, La Mantia I. Endoscopic surgical treatment for rhinogenic contact point headache: systematic review and meta-analysis. Eur Arch Otorhinolaryngol. 2021 Jun;278(6):1743-1753. doi: 10.1007/s00405-021-06724-6.

Added in introduction and limitations

23	Maniaci A, Lechien JR, La Mantia I, Calvo-Henriquez C, Iannella G, Locatello LG, et al. Effectiveness of submucosal turbinoplasty in refractory obstructive rhinitis: a prospective comparative trial. Eur Arch Otorhinolaryngol. https://doi.org/10.1007/s00405-022-07267-0 PMID: 35076746

Added in introduction

63	Caponnetto P, Emma R, Benfatto F, Ferlito S, Gulino A, Maniaci A, Lechien JR, Ingrassia A, Cocuzza S, Polosa R. Saccharin test: Methodological validation and systematic review of the literature. Ear Nose Throat J. 2021 Dec 16:1455613211064044. doi: 10.1177/01455613211064044. Epub ahead of print. PMID: 34915769.

Added in method

24	Cocuzza S, Maniaci A, Di Luca M, La Mantia I, Grillo C, Spinato G, Motta G, Testa D, Ferlito S. Long-term results of nasal surgery: comparison of mini-invasive turbinoplasty. J Biol Regul Homeost Agents. 2020 May-Jun;34(3):1203-1208. doi: 10.23812/19-522-L-4. PMID: 32640780.

Added in introduction

12	Maza-Solano J, Biadsee A, Sowerby LJ, Calvo-Hernández C, Tucciarone M, Rocha T, Maniaci A, Saibene AM, Chiesa-Estomba CM, Radulesco T, Metwaly O, Lechien JR, Alobid I, Locatello LG. Chronic rhinosinusitis with nasal polyps management in the biologic therapy era: an international YO-IFOS survey. Eur Arch Otorhinolaryngol. 2023 May;280(5):2309-2316. doi: 10.1007/s00405-022-07762-4. Epub 2022 Dec 1. PMID: 36454385.

Added in introduction

**Reviewer #2:** This is a systematic review article on oral Chinese herbal medicine after the treatment of sinusitis, which fully reflects the major advantages of traditional Chinese medicine in the treatment of chronic diseases.

**Comment 12.**

Introduction: What is the basis for the use of Chinese herbal medicine? What do common Western medical treatments include?

**Response 12.**

Thank you for this comment.

We have expanded the section on CHMs in the introduction (L111):

‘The Chinese guidelines for the diagnosis and treatment of CRS have included Chinese herbal medicines (CHMs) based on syndrome differentiation as additional treatment options [32, 42]. The CHMs can be administered orally and/or nasally [43, 44].

A review of various types of herbal medicines for CRS reported improvements in symptoms [36]. A systematic review of 34 studies of oral and/or nasal CHMs for acute or chronic RS, without surgery, found improvements in symptoms, sinus imaging and measures of mucociliary clearance [43].’

We have added a section on the Western medical treatments in the introduction (L81):

‘The conventional pharmacological management of CRS usually involves intranasal steroids, nasal saline irrigation and/or oral antibiotic therapy during exacerbations, with allergen avoidance in people with allergies [2, 3]. Biologics have recently been introduced [12].’

**Comment 13.**

The author should make appropriate additions in the background section. What are the advantages of Chinese herbal medicine and can be properly supplemented? The authors have published a systematic review on the treatment of chronic sinusitis with traditional Chinese medicine last year, which can be used for comparative analysis.

**Response 13.**

Thank you for these comments.

We have expanded the section on CHMs in the introduction (L111):

‘The Chinese guidelines for the diagnosis and treatment of CRS have included Chinese herbal medicines (CHMs) based on syndrome differentiation as additional treatment options [32, 42]. The CHMs can be administered orally and/or nasally [43, 44].

We added additional information on that systematic review in the introduction section (also see also Response 12 above) (L116).

‘A systematic review of 34 studies of oral and/or nasal CHMs for acute or chronic RS, without surgery, found improvements in symptoms, sinus imaging and measures of mucociliary clearance [43].’

We have added a comparison of the reviews in the discussion section (L572):

‘In comparison with the results of the systematic review of oral CHMs for RS [43], BYTQK was used in multiple studies included in both reviews. The two most frequent formulae from the earlier review, *Bi yuan shu kou fu ye* (BYSKFY) and its capsule form *Bi yuan shu jiao nang* (BYSJN), which were tested in four studies, and *Bi dou yan kou fu ye* (BDYKFY), which was tested in three studies, did not appear as interventions for CRS post-surgery. Future studies could consider testing these CHMs.’

**Comment 14.**
Methods: The authors do not elaborate exclusion criteria. The diagnostic criteria should be specific for the diagnosis of chronic sinusitis in the population with or without nasal polyps, as there is a significant difference in postoperative outcomes between the two, and this information should be reflected in the table.

**Response 14.**

Thank you for these comments.

We have added additional details of the exclusion criteria in the method section (L136-141):

‘Retrospective studies were excluded.’

‘Participants were adults and/or children diagnosed with sinusitis or rhinosinusitis with or without nasal polyps who had received surgery for their condition. Studies were excluded when any participants had fungal RS or non-RS nasal conditions, the condition was RS but not during post-surgical recovery, and/or the interventions included nasal CHMs.’

We have added the numbers of participants with polyps, dosages and the Chinese syndromes to Table 1.

We have added the diagnostic criteria, Chinese and Western, used in each study to S2 Table.

We have added post-hoc sensitivity analyses for presence of nasal polyps CRSwNP, or absence of polyps (CRSsNP) to the Method section (L186):

‘Post-hoc sensitivity analyses included presence or absence of nasal polyps and Chinese medicine syndrome.’

The results of these sensitivity analyses have been added to the results section of the manuscript and S6–S12 Tables.

**Comment 15.**

*Xin qian gan ju tang* (XQGJT) does not appear in Table 1, the author needs to check carefully.

**Response 15.**

We apologise for the typographical error in Table 1. We have changed the acronym from SQGJT to XQGJT.

See Table 1. Zhang JY 2019.

**Comment 16.**

According to the literature review, Biyuanshu oral liquid and sinusitis oral liquid are also important preparations with good curative effects. Please explain why experimental studies of these interventions have not been included. What exactly does the purified compound represent as defined by the author. This does not seem to be a reason to exclude "Biyuanshu oral liquid and sinusitis oral liquid".

**Response 16.**

Thank you for these comments.

By ‘purified compound’ we refer to single chemical entities (e.g., berberine), not herbal extracts. We have revised this to ‘single chemical compound’ to make the meaning clearer (L144). We did not exclude any studies for this reason.

In the previous review of RCTs of oral CHM for sinusitis (no surgery), the formula *Bi yuan shu* oral liquid BYSKFY (including its capsule form BYSJN) appeared 4 times and the formula sinusitis oral liquid (BDYKFY) (*Bi dou yan kou fu ye*) appeared 3 times. The reviewer is correct that these are important formulae for sinusitis. However, in the comprehensive searches of RCTs for oral CHM used post-surgery, these two formulae did not appear. We have not excluded them.

It is interesting that these two common CHMs had not been investigated post-surgery. We thank the reviewer for bringing this to our attention, and we have noted this point in the discussion (L572).

‘In comparison with the results of the systematic review of oral CHMs for RS [43], BYTQK was used in multiple studies included in both reviews. The two most frequent formulae from the earlier review, *Bi yuan shu kou fu ye* (BYSKFY) and its capsule form *Bi yuan shu jiao nang* (BYSJN), which were tested in four studies, and *Bi dou yan kou fu ye* (BDYKFY), which was tested in three studies, did not appear as interventions for CRS post-surgery. Future studies could consider testing these CHMs.’

**Comment 17.**

Results Section: The information provided by the authors is very comprehensive, but a collated table containing all the included articles (most of the basic information) is necessary to help the reader quickly capture the key information in the reading.

**Response 17.**

Thank you for these comments.

We have added data on polyps, dosage, and Chinese medicine syndrome to Table 1.

We added diagnostic criteria, Western and Chinese if available, to S2 Table.

If we merged Table 1 with the large S2 Table, the resultant table would wrap over multiple pages, so we have retained the ingredients in S2 Table.

**Comment 18.**

I feel it is very necessary to supplement a comparison of effectiveness (under the uniform evaluation criteria), which will intuitively reflect the effectiveness of treatment modalities.

**Response 18.**

Thank you for this comment.

In the revised version we have added separate analyses for studies in which all participants had the same diagnosis, CRSwNP or CRSsNP., for each outcome measure.

To the Results section we have added (L523)

‘In the pooled results for CRSwNP, there were significant differences between groups in favour of the CHM groups for VAS-TNS, LK and MTR. For CRSsNP, there were significant differences in favour of the CHM groups for LK, MTT and MTR, but not VAS-TNS. For the other outcomes, there were insufficient data.’

**Comment 19.**

Discussion section
It may be appropriate to add a section on modern pharmacological studies of drugs.

**Response 19.**

Thank you for this comment. It is an important topic but a comprehensive section on the experimental studies that have been conducted on the frequent herbal ingredients of the CHM would require many pages and is beyond the scope of this review.

Therefore, we have included a short summary of pharmacological actions at the end of the Discussion section as follows (L579):

‘When the herbal ingredients were compared, the most frequent ingredients in the formulae from the previous review were the same as in this review: *Magnolia biondii* (*xin yi*), *Angelica dahurica* (*bai zhi*), *Xanthium sibiricum* (*cang er zi*), and *Scutellaria baicalensis* (*huang qin*). In experimental studies, extracts of each of these four plants have been reported to demonstrate anti-inflammatory, anti-allergic and antioxidant effects. In addition, antimicrobial effects have been reported for *Angelica dahurica, Xanthium sibiricum,* and *Scutellaria baicalensis,* while antiviral effects have been shown for extracts of *Xanthium sibiricum* and *Scutellaria baicalensis* [76]. These pharmacological actions of the herbs included in multiple CHMs may provide insight in to how these CHMs exerted the clinical effects reported in the RCTs.’

**Comment 20.**

The biggest limitation of this study is that the types of Chinese medicine preparations differ, and the authors should add this note. It is hoped that more high-quality RCTs with the same prescription will be supplemented in the future.

**Response 20.**

Thank you for this comment.

We have added the following section in the limitations (L625):

‘Although many CHMs contained common ingredients, there was considerable variation in the test interventions, with only three CHM formulae (BYTQK, HQHST and SLBZS) being tested in multiple (2 or 3) studies. The diversity in the CHMs was a likely source of heterogeneity in the meta-analyses. More high-quality RCTs are needed for previously tested CHMs to provide rigorous assessments of their effects.’

**Additional changes**

The MS and supplementary have been checked, edited, and corrected throughout for: spelling, nomenclature, acronyms, punctuation, and layout. Sentences have been shortened where possible and redundancy has been removed. These changes appear in the track changes versions.

END
